# Supplementary material for: Partial Biodegradable Blend with High Stability against Biodegradation for Fused Deposition Modeling
Source: Polymers (Basel). 2022 Apr 11;14(8):1541. doi: 10.3390/polym14081541 (PMC9027655; doi:10.3390/polym14081541)
Supplement: Supplementary file 1 [file polymers-14-01541-s001.zip › polymers-1656305-supplementary.pdf]

polymers MDPI

Article

Supplementary File S1: Minitab analysis for Soil biodegradation

Partial Biodegradable Blend with High Stability against Biodegradation for Fused Deposition Modeling

Muhammad Harris [1,2,*], Hammad Mohsin [3], Johan Potgieter [1], Kashif Ishfaq [4], Richard Archer [5], Qun Chen [5], Karnika De Silva [6], Marie-Joo Le Guen [7], Russell Wilson [1] and Khalid Mahmood Arif [8]

[1] Massey Agrifood Digital Lab, Massey University, Palmerston North 4410, New Zealand; j.potgieter@massey.ac.nz (J.P.); r.wilson@massey.ac.nz (R.W.)
[2] Industrial and Manufacturing Engineering Department, Rachna College of Engineering and Technology, Gujranwala 52250, Pakistan
[3] Department of Polymer Engineering, National Textile University, Faisalabad 37610, Pakistan; mhammad@ntu.edu.pk
[4] Industrial and Manufacturing Engineering Department, University of Engineering and Technology, Lahore 54890, Pakistan; kashif.ishfaq@uet.edu.pk
[5] School of Food and Advanced Technology, Massey University, Palmerston North 4410, New Zealand; r.h.archer@massey.ac.nz (R.A.); q.chen2@massey.ac.nz (Q.C.)
[6] Faculty of Engineering, University of Auckland, Auckland 1023, New Zealand; k.desilva@auckland.ac.nz
[7] Scion, Rotorua 3046, New Zealand; mariejoo.leguen@scionresearch.com
[8] Department of Mechanical and Electrical Engineering, SF&AT, Massey University, Auckland 0632, New Zealand; k.arif@massey.ac.nz
* Correspondence: m.harris@massey.ac.nz

Citation: Harris, M.; Mohsin, H.; Potgieter, J.; Ishfaq, K.; Archer, R.; Chen, Q.; DeSilva, K.; Guen, M.-J.L.; Wilson, R.; Arif, K.M. Partial Biodegradable Blend with High Stability against Biodegradation for Fused Deposition Modeling. *Polymers* **2022**, *14*, 1541. https://doi.org/10.3390/polym14081541

Academic Editor: Abdel-Hamid I. Mourad

Received: 12 March 2022
Accepted: 7 April 2022
Published: 11 April 2022

Publisher's Note: MDPI stays neutral with regard to jurisdictional claims in published maps and institutional affiliations.

Copyright: © 2022 by the authors. Submitted for possible open access publication under the terms and conditions of the Creative Commons Attribution (CC BY) license (https://creativecommons.org/licenses/by/4.0/).

ANOVA analysis

The randomized multi-level general full factorial design of experiment (DoE) for the effects of soil degradation and in-process printing temperatures on tensile strength is as provided in Table S1.

Table S1. DoE for analysis of soil degradation effects on tensile strength.

StdOrder	RunOrder	PtType	Blocks	Bed temperature	Printing temperature	Soil Treatment	Tensile strength (MPa)
4	1	1	1	25	166	Treated	37.15
6	2	1	1	25	171	Treated	42.79104
8	3	1	1	55	161	Treated	39.53389
5	4	1	1	25	171	Non-treated	43.37669
15	5	1	1	85	166	Non-treated	32.49289
1	6	1	1	25	161	Non-treated	38.92701
18	7	1	1	85	171	Treated	38.850825
13	8	1	1	85	161	Non-treated	44.959735
17	9	1	1	85	171	Non-treated	43.10712
11	10	1	1	55	171	Non-treated	40.01403
7	11	1	1	55	161	Non-treated	42.99
9	12	1	1	55	166	Non-treated	37.71559
16	13	1	1	85	166	Treated	36.9
2	14	1	1	25	161	Treated	33.936205
3	15	1	1	25	166	Non-treated	36.12446
12	16	1	1	55	171	Treated	35.73578
10	17	1	1	55	166	Treated	37.063755
14	18	1	1	85	161	Treated	37.5

WORKSHEET 1

General Factorial Regression: Tensile strength (MPa) versus Bed temperature, Printing temperature, Soil Treatment

Factor Information

Factor	Levels Values
Bed temperature	3 25, 55, 85
Printing temperature	3 161, 166, 171
Soil Treatment	2 Non-treated, Treated

Analysis of Variance

Source	DF	Adj SS	Adj MS	F-Value	P-Value
Model	13	191.185	14.7066	4.22	0.087
Linear	5	86.905	17.3810	4.99	0.072
Bed temperature	2	0.189	0.0944	0.03	0.973
Printing temperature	2	63.944	31.9720	9.18	0.032
Soil Treatment	1	22.772	22.7723	6.54	0.063
2-Way Interactions	8	104.280	13.0350	3.74	0.109
Bed temperature*Printing temperature	4	65.906	16.4764	4.73	0.081
Bed temperature*Soil Treatment	2	1.304	0.6521	0.19	0.836
Printing temperature*Soil Treatment	2	37.070	18.5351	5.32	0.075
Error	4	13.934	3.4836		
Total	17	205.120			

Model Summary

S	R-sq	R-sq(adj)	R-sq(pred)
1.86643	93.21%	71.13%	0.00%

Coefficients

Term	Coef	SE Coef	T-Value	P-Value	VIF
Constant	38.843	0.440	88.29	0.000	
Bed temperature					
25	-0.125	0.622	-0.20	0.850	1.33
55	-0.001	0.622	-0.00	0.999	1.33
Printing temperature					
161	0.798	0.622	1.28	0.269	1.33
166	-2.602	0.622	-4.18	0.014	1.33
Soil Treatment					
Non-treated	1.125	0.440	2.56	0.063	1.00
Bed temperature*Printing temperature					
25 161	-3.084	0.880	-3.51	0.025	1.78
25 166	0.521	0.880	0.59	0.585	1.78
55 161	1.621	0.880	1.84	0.139	1.78
55 166	1.149	0.880	1.31	0.262	1.78
Bed temperature*Soil Treatment					
25 Non-treated	-0.366	0.622	-0.59	0.588	1.33
55 Non-treated	0.273	0.622	0.44	0.684	1.33
Printing temperature*Soil Treatment					
161 Non-treated	1.526	0.622	2.45	0.070	1.33
166 Non-treated	-1.922	0.622	-3.09	0.037	1.33

Regression Equation

Tensile strength (MPa) = 38.843 - 0.125 Bed temperature_25 - 0.001 Bed temperature_55
+ 0.126 Bed temperature_85 + 0.798 Printing temperature_161
- 2.602 Printing temperature_166 + 1.803 Printing temperature_171
+ 1.125 Soil Treatment_Non-treated - 1.125 Soil Treatment_Treated
- 3.084 Bed temperature*Printing temperature_25 161
+ 0.521 Bed temperature*Printing temperature_25 166
+ 2.563 Bed temperature*Printing temperature_25 171
+ 1.621 Bed temperature*Printing temperature_55 161
+ 1.149 Bed temperature*Printing temperature_55 166
- 2.770 Bed temperature*Printing temperature_55 171
+ 1.463 Bed temperature*Printing temperature_85 161
- 1.670 Bed temperature*Printing temperature_85 166
+ 0.207 Bed temperature*Printing temperature_85 171
- 0.366 Bed temperature*Soil Treatment_25 Non-treated
+ 0.366 Bed temperature*Soil Treatment_25 Treated
+ 0.273 Bed temperature*Soil Treatment_55 Non-treated
- 0.273 Bed temperature*Soil Treatment_55 Treated
+ 0.093 Bed temperature*Soil Treatment_85 Non-treated
- 0.093 Bed temperature*Soil Treatment_85 Treated
+ 1.526 Printing temperature*Soil Treatment_161 Non-treated
- 1.526 Printing temperature*Soil Treatment_161 Treated
- 1.922 Printing temperature*Soil Treatment_166 Non-treated
+ 1.922 Printing temperature*Soil Treatment_166 Treated
+ 0.395 Printing temperature*Soil Treatment_171 Non-treated
- 0.395 Printing temperature*Soil Treatment_171 Treated

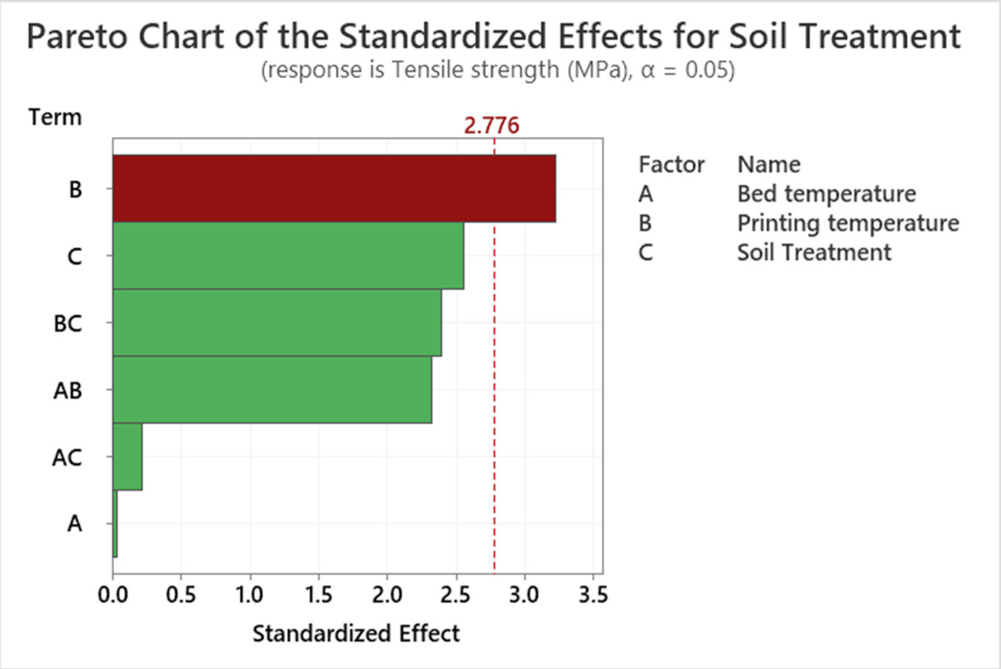

Figure S1. Pareto chart for the first trial of soil treatment samples.

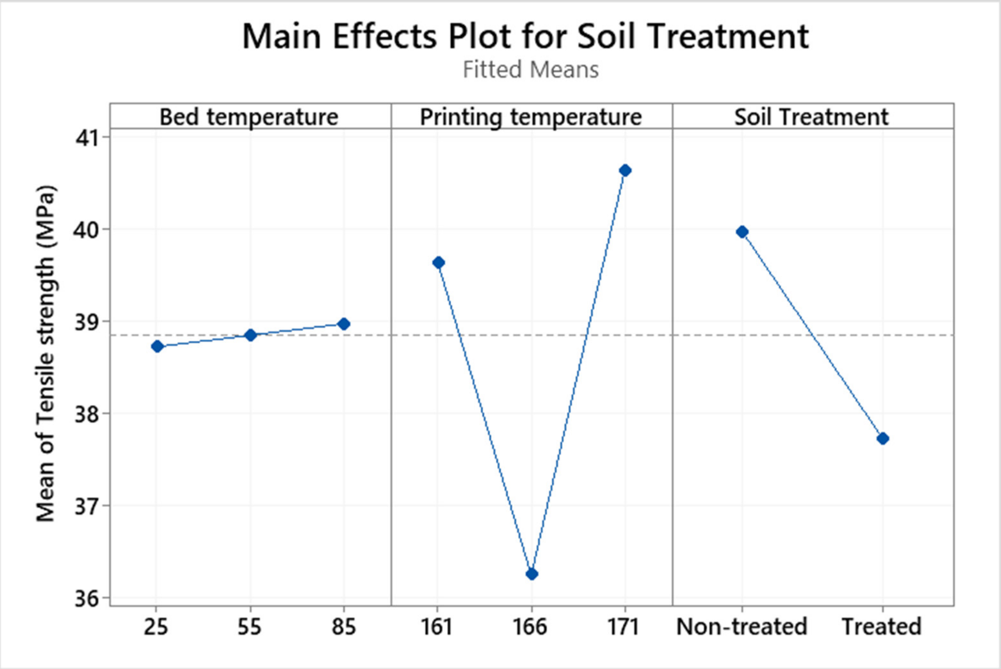

Figure S2. Main effects plots for soil treatment samples.

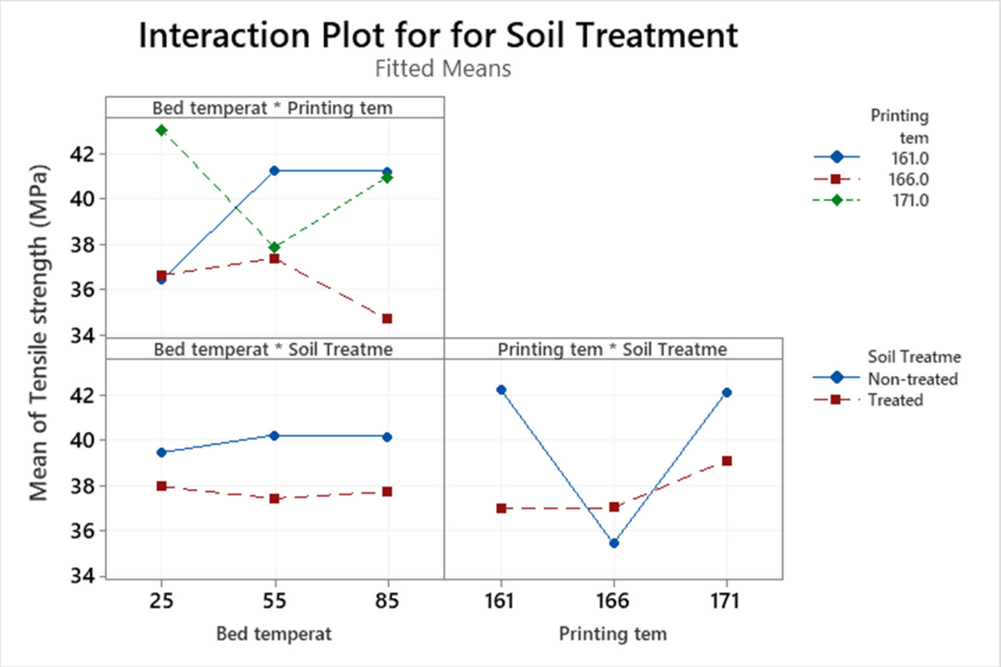

Figure S3. Interaction plots for soil treatment samples.
